# Harnessing the Power of Plants: A Green Factory for Bioactive Compounds

**DOI:** 10.3390/life13102041

**Published:** 2023-10-11

**Authors:** Jianfeng Xu

**Affiliations:** 1 Arkansas Biosciences Institute, Arkansas State University, Jonesboro, AR 72401, USA; jxu@astate.edu; Tel.: +1-870-680-4812; 2 College of Agriculture, Arkansas State University, Jonesboro, AR 72401, USA

The plant kingdom has long been revered for its complex biochemical pathways, which give rise to an incredible array of bioactive compounds. These small-molecule compounds can be generally classified as alkaloids, terpenoids/terpenes, and phenolic compounds. Many of these compounds have proven to be of great value to human beings as pharmaceuticals or nutraceuticals, as they exhibit antioxidant, antitumor, anti-inflammatory, and antibacterial effects and also show potential functions in immunomodulation, neuroprotection, and antiallergy. Moreover, the agricultural sector has benefited significantly from the use of bioactive compounds in the form of agrochemicals. Pesticides derived from plant-based terpenoids, for instance, help safeguard crops from pests and diseases while minimizing environmental harm. In addition to native compounds, plants can also be genetically engineered to produce valuable recombinant proteins (termed “molecular pharming”) for therapeutic and industrial applications (e.g., cytokines, blood proteins, antibodies, vaccines, and industrial enzymes). The driving forces behind the rapid growth of plant-based biofactories include their relatively low production cost, product safety, and easy scale-up process.

This Special Issue of Life, entitled “*Plants as a Promising Biofactory for Bioactive Compounds*,” showcases the latest research on the utilization of plant biofactories for producing compounds with multiple biological effects, including antioxidant, antitumor, antidiabetic, anti-inflammatory, antimicrobial, antialzheimer, antihemolytic, neuroprotective, and pesticide activities. The 17 articles contained within this Special Issue, of which 13 are research papers and 4 are reviews, encompass a broad spectrum of research, ranging from fundamental studies elucidating the biosynthetic pathways of bioactive compounds to applied research harnessing the biotechnological potential of plant-based production systems. They collectively highlight the incredible potential of plants as green factories for producing bioactive compounds.

One recurring theme in these papers is the extraction, isolation, and structural characterization of existing or new bioactive compounds with nutraceutical and therapeutic potential from medicine plants. Advanced analytic tools, such as LC-HR/MS, GC/MS, and GC-FID, were commonly exploited to determine the bioactive compound profile or phytochemical composition in plant extracts. The pharmaceutical or nutraceutical activities of the separated compounds or crude plant extracts were analyzed in vitro or in vivo. The paper by Murthy and Dewir’s group determined the phytochemical composition of different parts of *Andrographis macrobotrys* Nees and demonstrated the importance of *A. macrobotrys* as a source of medicine and antioxidants [1]. Karagecili and colleagues analyzed the chemical profiling of peel and seed extracts of Zivzik Pomegranate (*Punica granatum*) via LC-MS/MS and determined the antioxidant, antialzheimer, antidiabetic, antiglaucoma, and antimicrobial effects of different phenolic and flavonoid compounds [2]. Inspired by the remarkable antitumor potential of many plant secondary metabolites, Gomez-Flores’ team conducted an assay on of the effects of methanol extracts from 15 selected Mexican medicinal plants on the growth inhibition of mouse lymphoma cells, the toxicity and proliferation of human peripheral blood mononuclear cells, and their antioxidant, hemolytic, and antihemolytic activities [3]. Their results validate the possibility of identifying bioactive compounds showing antitumor, antioxidant, and antihemolytic activity from medicinal plants. The paper by Hassan and colleagues presented the first metabolomic profiling of the halophyte *Agathophora alopecuroides*, a potential antidiabetic plant, using the LC-HRMS/MS technique [4]. It is worth mentioning that a molecular docking technique was adopted for this study to highlight the bioactive compounds, of which two alkaloids and four flavonoids were identified as being responsible for the observed antidiabetic activity.

In addition to analyzing the broad spectrum of compounds in medicinal plants, five papers focus on two specific types of bioactive compounds: essential oils and phenolic compounds. In a study conducted by Gulcin’s group, the antioxidant and antidiabetic properties of essential oil from cinnamon (*Cinnamomum zeylanicum*) leaves were evaluated and investigated for the first time. Furthermore, the inhibitory effects of cinnamon oil on enzymes associated with various metabolic diseases were also determined [5]. In another study, El-Kased and EI-Kersh analyzed the GC–MS profiling of eight natural essential oils and demonstrated their antimicrobial effects and their beverage preservative actions [6]. For phenolic compounds, Vaitiekūnaitė group’s research indicated that the accumulation of phenolic and antioxidant compounds in *Quercus robur* Bark diverges based on tree genotype, phenology, and the extraction method used [7]. Olennikov’s team profiled and quantified the phenolic compounds of leaf extracts of two Caucasian blueberries, namely *Vaccinium myrtillus* L. and *Vaccinium arctostaphylos* L., via HPLC–PDA–ESI–tQ–MS and showed their neuroprotective and antioxidant potential [8]. Similarly, the study by Hussain and colleagues demonstrated that polyphenols extracted from *Salvadora oleoides* Decene. and *Salvadora persica* L. fruit and aerial parts could potentially be used as antioxidant, analgesic, and anti-inflammatory agents [9]. Other than having applications as nutraceuticals and therapeutics, plant-derived compounds can also be used as pesticides. In their study, Klasson and colleagues extracted aconitic acid from sweet sorghum syrup and used it as a nematicide [10]. The extract was found to effectively reduce the motility of the parasitic *Meloidogyne incognita* and cause a high mortality of the nematode.

The utilization of advanced biotechnological methods such as metabolic engineering, genome editing, and plant tissue culture have allowed researchers to fine-tune the biosynthetic pathways of plants, thereby improving the yields and quality of bioactive compounds. There are two research papers in this Special Issue that tackle this research area. An excellent paper by Qi’s group explored the characterization, evolution, expression patterns, and gene function of BAHDs in *Lithospermum erythrorhizon* (LeBAHDs) via bioinformatics and transgenic analysis [11]. The plant BAHD acyltransferase family has been well known to acylate plant metabolites and participate in plant secondary metabolic processes. The results from the study by Qi’s group revealed that the overexpression of LeBAHD1 in *L. erythrorhizon* hairy roots significantly increased the content of acetylshikonin and the conversion rate of shikonin to acetylshikonin, whereas the CRISPR/Cas9-based knockout of LeBAHD1 in hairy roots displayed the opposite trend. This study not only confirmed the in vivo function of LeBAHD1 in the biosynthesis of acetylshikonin but also provided new insights into the biosynthetic pathway of shikonin and its derivatives. In another study on improving essential oil production in aromatic plants, Devlin’s group found that the addition of Arbuscular Mycorrhizal Fungi (AMF) profoundly influenced terpene synthase expression in six rosemary cultivars without impacting plant growth [12]. Their findings demonstrated the potential for the use of AMF in the improvement of aroma in culinary herbs within a commercial setting.

Apart from the extensive body of research dedicated to exploring bioactive small molecules derived from plants, a notable study by Dr. Guerineau reported the successful production of recombinant human gastric lipase by *Arabidopsis thaliana* root culture while also providing a comprehensive characterization of the enzyme’s properties [13]. This investigation showcases the application of molecular pharming in plants as a means to synthesize recombinant proteins, which are big molecules.

In addition to these noteworthy research articles, this Special Issue also contains four review papers that discuss the pharmacological applications of plant-derived bioactive compounds. Emran and colleagues discussed the potential of naringin and naringenin polyphenols as therapeutics for neurodegenerative diseases such as Alzheimer’s disease and Parkinson’s disease, as well as other neurological conditions such as anxiety, depression, and chronic hyperglycemic peripheral neuropathy [14]. Cruz-Cansino and colleagues focused on the exploitation of by-products from fruits and vegetables. Specifically, the applications and pharmacological properties of bioactive compounds from cactus pear (*Opuntia* spp.) peel were discussed [15]. In addition to bioactive secondary metabolites, a review paper by Havrlentová’s group focused on other types of bioactive molecules from plants, namely β-D-glucan, a cell wall polysaccharide [16]. The comprehensive introduction to this bioactive biomolecule from Poales expands our understanding of the characteristics, functions, and applications of this cell wall polysaccharide and opens new avenues for future research and advancements. Finally, in light of the recent outbreak of COVID-19, caused by severe acute respiratory syndrome coronavirus 2 (SARS-CoV-2), which had a profound impact on public health and the global economy, Xu’s group presented a comprehensive review on the latest advancements in plant-derived antiviral medicines, including both small-molecule compounds and recombinant therapeutics used to combat COVID-19 [17]. Particularly, the unprecedented opportunity of molecular pharming in plants for developing vaccines, antibodies, and other biologics against COVID-19 was demonstrated. This review sheds light on the future development of plant sources of antiviral medicines for treating COVID-19 and other viral infections.

In a world where the exploration of sustainable sources for bioactive compounds has become increasingly significant, the potential of plants as biofactories has garnered substantial attention. This collection of 17 papers represents a pivotal step forward in our understanding of how plants can serve as potent sources of bioactive molecules with diverse applications. The interdisciplinary nature of these contributions underscores the collaborative efforts of botanists, chemists, biotechnologists, and pharmacologists, all working towards a common goal: harnessing the power of plants to produce valuable compounds.
